# A Network Approach to Analyzing Highly Recombinant Malaria Parasite Genes

**DOI:** 10.1371/journal.pcbi.1003268

**Published:** 2013-10-10

**Authors:** Daniel B. Larremore, Aaron Clauset, Caroline O. Buckee

**Affiliations:** 1 Department of Epidemiology, Harvard School of Public Health, Boston, Massachusetts, United States of America; 2 Center for Communicable Disease Dynamics, Harvard School of Public Health, Boston, Massachusetts, United States of America; 3 Department of Computer Science, University of Colorado, Boulder, Colorado, United States of America; 4 BioFrontiers Institute, University of Colorado, Boulder, Colorado, United States of America; 5 Santa Fe Institute, Santa Fe, New Mexico, United States of America; Emory University, United States of America

## Abstract

The *var* genes of the human malaria parasite *Plasmodium falciparum* present a challenge to population geneticists due to their extreme diversity, which is generated by high rates of recombination. These genes encode a primary antigen protein called PfEMP1, which is expressed on the surface of infected red blood cells and elicits protective immune responses. *Var* gene sequences are characterized by pronounced mosaicism, precluding the use of traditional phylogenetic tools that require bifurcating tree-like evolutionary relationships. We present a new method that identifies highly variable regions (HVRs), and then maps each HVR to a complex network in which each sequence is a node and two nodes are linked if they share an exact match of significant length. Here, networks of *var* genes that recombine freely are expected to have a uniformly random structure, but constraints on recombination will produce network communities that we identify using a stochastic block model. We validate this method on synthetic data, showing that it correctly recovers populations of constrained recombination, before applying it to the Duffy Binding Like-α (DBLα) domain of *var* genes. We find nine HVRs whose network communities map in distinctive ways to known DBLα classifications and clinical phenotypes. We show that the recombinational constraints of some HVRs are correlated, while others are independent. These findings suggest that this micromodular structuring facilitates independent evolutionary trajectories of neighboring mosaic regions, allowing the parasite to retain protein function while generating enormous sequence diversity. Our approach therefore offers a rigorous method for analyzing evolutionary constraints in *var* genes, and is also flexible enough to be easily applied more generally to any highly recombinant sequences.

## Author Summary

The human malaria parasite kills nearly 1 million people each year globally. Frequent genetic exchange between malaria parasites creates enormous genetic diversity that largely explains the lack of an effective vaccine for the disease. Traditional phylogenetic tools cannot accommodate this type of diversity, however, and rigorous analytical tools capable of making sense of gene sequences that recombine frequently are still lacking. Here, we use network techniques that have been developed by the physics and network science communities to analyze malaria parasite gene sequences, allowing us to automatically identify highly variable mosaic regions in sequence data and to derive the network of recombination events. We apply our method to seven fully-sequenced parasite genomes, and show that our method provides new insights into the complex evolutionary patterns of the parasite. Our results suggest that the structure of these sequences allows the parasite to rapidly diversify to evade immune responses while maintaining antigen structure and function.

## Introduction

The human malaria parasite *Plasmodium falciparum* causes approximately 1 million deaths each year, primarily in young children in sub-Saharan Africa [1]. In endemic regions, individuals develop clinical immunity to severe disease in childhood, but continue to suffer malaria infections and mild illness throughout their lifetimes. This epidemiological pattern is poorly understood, but appears to be caused by the gradual acquisition of a large repertoire of antibodies following sequential exposure to different parasite proteins [2]–[5]. The main candidate for eliciting protective antibodies is the parasite-derived antigen PfEMP1 (*P. falciparum* erythrocyte membrane protein 1), encoded in each parasite genome by a large *var* gene family and expressed during infection on the surface of infected red blood cells in a process of antigenic variation [2], [6]–[9]. Extremely rapid recombination among *var* genes generates enormous diversity and complex mosaic structures among these sequences [10]–[14], and recent field studies have uncovered seemingly limitless *var* gene diversity in Africa [15]. Superinfection with multiple clones is extremely common, and recombination can occur during meiosis in the mosquito, as well as between *var* genes on different chromosomes of a single parasite during asexual reproduction [14]. However, these observations are at odds with the rapid acquisition of antibodies to common PfEMP1 variants that are associated with disease [16], as well as the finding that parasites from different continents share identical sequence blocks despite millions of years of evolutionary separation [17].

The highly recombinant structure of *var* genes precludes the use of standard phylogenetic tools, and the processes generating this paradoxical relationship between parasite genetic structure and the epidemiology of infection and disease remain unclear [13], [18], [19]. Statistically rigorous and scalable techniques to analyze evolutionary relationships between sequences generated through frequent recombination are lacking. Classical phylogenetic analyses are designed to accommodate branching tree-like relationships between genes generated by mutation, and therefore require that highly recombinant regions, where evolutionarily distant sequences may share mosaics, are removed, ignored, or assumed to be absent [20]–[22]. Bockhorst *et al.* introduced an approach to understanding the most conserved group of *var* genes based on a segmentation analysis which divides a set of sequences into segments such that polymorphic sites in the same segment are strongly correlated, while nearby polymorphic sites are either weakly or not correlated [18], [23]. While segmentation analysis is useful to detect mutation-driven diversification following ancient recombination or geographic separation, particularly for subsets of more conserved *var* genes, it ultimately generates a tree-like relationship between genes and does not accommodate recent and ongoing recombination.

Networks provide a mathematical approach to representing and studying complex relationships between genes [24], and network-based techniques have produced valuable evolutionary insights for many organisms ranging from viruses to eukaryotes [25]. Attempts to introduce recombination within phylogenetic frameworks have led to specialized techniques that produce phylogenetic (or recombination) networks for small numbers of sequences when recombination rates are relatively low, but these also focus on conserved regions rather than providing insights into the recombinant regions [26] (for a review see [27]). On the other hand, ancestral recombination graphs have a strong theoretical foundation but lack efficient approximations that are required for rapid inference [28]. Networks have also been used to identify large-scale clusters of global gene sharing and exchange [29] and horizontal gene transfer of the plasmid resistome [30], as well as differentiating horizontal and vertical flow of information in [31], [32]. In these approaches, all-to-all BLAST scores are calculated and thresholded for a set of sequences, and the resulting network is generally analyzed visually to assess large-scale structure [22], [25], [29]–[32]. These analyses, however, rely on *ad hoc* parameter choices, uncontrolled assumptions, or prior knowledge of target clustering. And, while potentially useful for hypothesis generation, a reliance on alignment scores contains an implicit model for sequence mutation and substitution that is not justified for highly recombinant *var* gene sequences. We have previously taken a network approach to analyze clinical *var* gene domains using short position-specific sequences [13]. Although this approach uncovered distinctive structuring, with clustering that reflected previous *var* gene classification schemes, it lacked a solid theoretical basis and more importantly it was not generalizable to other domains and genes.

Here we take a more sophisticated approach, applying rigorous community detection methods that have primarily been developed in the physics, statistics, and network science literature, to construct and analyze recombinant gene networks in general, and *var* gene networks in particular. We apply our technique to previously published and annotated sequences of the *var* Duffy Binding Like-α (DBLα) domain [18], [33], which unlike other domains is found in almost all *var* genes sequenced. We show that networks constructed from different mosaic regions across the domain vary widely in their community structure, uncovering a new layer of micromodularity among *var* genes. Our results imply a lack of coupled evolution within even a single domain. At the same time, clear structures within networks correspond well, and differentially, to previously published classifications that have been linked to disease phenotypes. This structuring therefore provides a mechanism to generate vast diversity while maintaining protein structure and function, reconciling the paradoxical observations of both common serological responses and almost limitless *var* sequence diversity.

## Methods

### *Plasmodium falciparum var* gene data

We analyze 307 amino acid sequences from the DBLα domain of the *var* genes of seven *P. falciparum* isolates published in [18]. PfEMP1 antigens exhibit modular structures, characterized by between two and nine DBL and CIDR (Cysteine-Rich Interdomain Region) domains [18], [34]–[36]. While there are many different classes of these domains, indexed by α, β, etc., the N-terminal region of the protein almost always begins with a DBLα and CIDRα pair, each of which has been implicated in the binding of infected red blood cells to various host receptors as well as different disease pathologies. To highlight the diversity and mosaicism of the DBLα domain we first apply standard phylogenetic approaches to the 307 sequences. Sequence length prior to alignment was widely distributed between 357 and 473 amino acids with median 420 and mode 398. Pair-wise alignments using the standard tool MUSCLE [16], [37] averaged 5.6% (or 23) gaps. Due to the presence of highly variable regions, a multiple alignment required an implausibly large number of gap insertions, yielding an aligned length of 743. The remarkable diversity in DBLα sequences is further illustrated in the unresolved nature of a phylogenetic tree built from such an alignment (Figure S3).

### General approach

**Figure 1:**
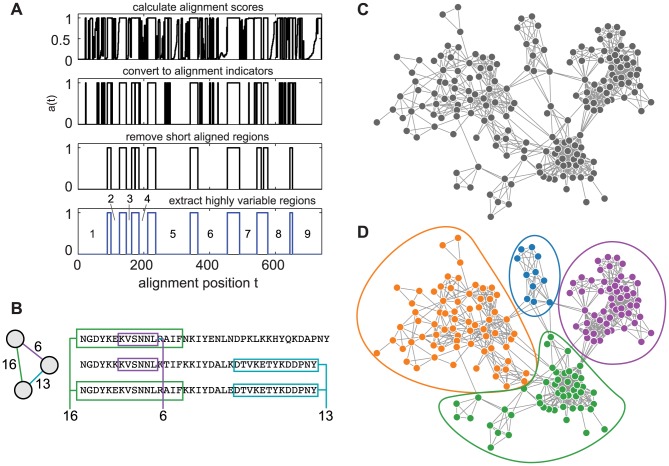
Pictorial overview of sequence analysis method. (A) Starting from a multiple alignment of the domain of interest, four steps are taken to identify highly variable regions (HVRs), as described in the text. We show the HVR identification process for the 307 DBLα sequences from [18]. (B) For each HVR, a network is made in which each sequence is a node, and a link connecting two nodes corresponds to a shared sequence block. (C) The set of pairwise connections above the noise threshold defines a complex network representing recent recombination events. (D) Communities are inferred directly from this network using a probabilistic generative model. Steps B,C, and D are repeated for each of the HVRs identified in step A.

Instead of using alignments to identify evolutionary signals contained in highly conserved regions, we use them to identify and remove conserved regions in order to focus on recombinant mosaic sequences. The method uses three steps, which we motivate here, and define in detail in the next sections: (i) Identify highly variable regions (HVRs) across all sequences. (ii) Compare sequences pair-wise within each HVR, generating a distinct block-sharing network for each region. (iii) Statistically identify communities in each network, which will represent groups of *var* genes that recombine more frequently with each other than with genes from other communities. These general steps are illustrated in Figure 1.

To identify HVRs, we use the basic premise of an alignment as a starting point: highly variable regions will require gap insertions in order to find an alignment. In contrast with other methods used to identify regions of conservation by discarding poorly-aligned stretches [38], our explicit goal is to find contiguous poorly-aligned regions, since these are likely to be mosaics resulting from recombination. After identifying HVRs, instead of constructing trees, we generate a complex network for each HVR using an alignment-free process, where each vertex is a sequences and two sequences are connected if they exhibit a pattern of recombination. The structure of this network reflects the constraints and extents of the recombination process. Since any two genes may recombine in the absence of constraints on recombination, deviations from a random network represent structured recombination, function, or evolution between *var* gene communities. To analyze network structures, we use a community-detection approach that can identify the patterns produced by constrained recombination, by fitting a generative model called a degree-corrected stochastic block model [39] to the network data. The degree-corrected stochastic block model identifies communities by picking out non-random patterns in the network connections making it an appropriate choice among myriad community detection methods. Each step is described in detail below.

### Detailed approach

In the first of three steps, we take a set of amino acid sequences and identify highly variable regions (HVRs). Starting from a multiple alignment, each aligned position *t* is first assigned an alignment score representing the fraction of input sequences that are aligned at that position (i.e. not gap insertions). This score is used to calculate an alignment indicator *a(t)* such that when all sequences align with no gaps at position *t*, *a(t) = 1*; if there are any gaps, *a(t) = 0*. We then identify regions where the sequences align for *G* or more consecutive positions, that is, *a(t) = 1* for *G* or more consecutive *t*. These well aligned regions will serve as separators between HVRs. We choose *G* sufficiently short, based on a simple null model of sequences (SI1), that any highly conserved block of significant length is removed from network construction since it would obscure patterns of recombination. Thus, the HVRs will be the regions in between the conserved regions that we have just identified. However, very short HVRs will have so few amino acids once gaps are removed in subsequent steps that they are unlikely to reflect the mosaicism in which we are interested. So, we define a minimum HVR size *H*, discarding those that are shorter. These steps are illustrated in Figure 1A.

**Figure 2:**
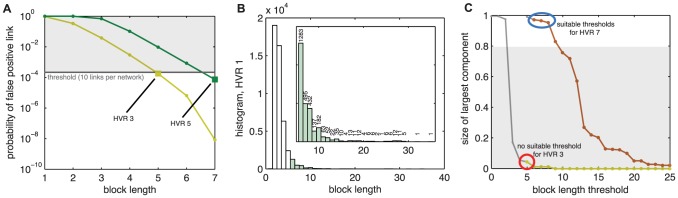
Choice of link noise threshold. Choosing a noise threshold requires balance between two competing requirements for correctly identifying network communities: minimize the number of incorrectly placed links, yet retain as many correctly placed links as possible to satisfy the network connectivity requirements of the community detection method. (A) The probability of two sequences sharing a block while not actually being related decreases as block length increases, modeled in S1. Each HVR's length and composition are taken into account separately (colored lines). Choosing a tolerance for false positives (grey line) specifies a minimum retained block length; since blocks are of integer length, the next largest integer is the minimum retained block length (squares). Curves for HVRs 3 and 5 are plotted, for which we would select thresholds of five and seven, respectively. Curves for all nine HVRs are shown in Fig. S6A. (B) For a choice of threshold 6 for HVR 1, the histogram of HVR 1 block lengths shows that a vast majority of the blocks are below the threshold (white bars) and that the retained blocks are widely distributed (green bars, inset). (C) Networks are fragmented as the block length threshold is increased and more links are discarded. The relationship between the size of the largest component and block length threshold is shown for the least-connected (HVR3) and most-connected (HVR7) networks. Some thresholds allow too many false positives, as described in panel A (grey lines), yet other thresholds fragment the network too much for reliable community detection (shaded region). Those points that are plotted in color above the shaded region are both sufficiently error-free and well connected that we may reliably infer network communities. For HVRs 2–4, even the most permissive false positive threshold results in a network that is too fragmented for community detection (red circle). Curves for all nine HVRs are shown in Figure S6B.

In the second step, we take each HVR and produce an unweighted and undirected recombination network. Each node represents a sequence, and each link represents a shared sequence block, indicating a recombinant relationship between the two sequences. Before comparing sequences, all gap insertions from the alignment process are removed. First, we create a weighted network in which the weight of each link is the length of the longest substring shared between the sequences it connects (Figure 1B). The result is an all-to-all undirected and weighted network for each HVR. Thus, for sequences with multiple HVRs there will be multiple networks. Next, we convert each all-to-all weighted network into a sparse and unweighted network by discarding links with weight below a threshold, and removing weights from the remaining links (Figure 1C). Here we choose the threshold in a way that controls the number of false positive links that may have arisen by chance, as shown in Figures 2A and 2B. The method for computing a noise threshold is based on a null model for randomly assembled sequences using the properties of each HVR, and not derived from network properties. Thus, depending on the confidence one wishes to have in the validity of the network's links, a threshold may be computed from a selected tolerable error rate. Derivation of the function used for this computation is included in Text S1.

In the third and final step, we detect recombination communities, by taking an unweighted and undirected HVR network and applying a degree-corrected stochastic block model [39] to identify community structures, illustrated by Figure 1D. This model takes as its input an unweighted, undirected network and the number of communities *k* for which it should find a maximum likelihood fit, and provides as an output a list of which nodes belong to which of the *k* communities, also referred to as a *partition*. (Derivation and maximization of the likelihood function are discussed at length in Ref. [39] and efficient code has been made publicly available by Karrer and Newman.) Because previous classifications included between three and six types, we inferred community structures for *k = 3* to *k = 6*. Each HVR network may have different community structure, similar to how in a standard approach, different loci may generate different phylogenetic trees. However, clades in trees represent distinct branches in an evolutionary history, while HVR network communities represent distinct clusters of ongoing recombination. Multiple trees may be combined to produce a consensus tree, but HVR networks show no clear consensus. We compare our resulting network communities to previous analyses of *var* gene sequence groups. Weights are removed from the network prior to community detection for two reasons. First, it is unclear by what principle differences in weights should be interpreted when defining communities. Second, the problem of correctly inferring degree-corrected stochastic block model community structure in sparse and weighted networks is currently unsolved. For these reasons, we interpret each network link as evidence of some recombinant or hereditary history and treat them equally by unweighting networks prior to community detection. We validate this approach carefully as follows.

**Figure 3:**
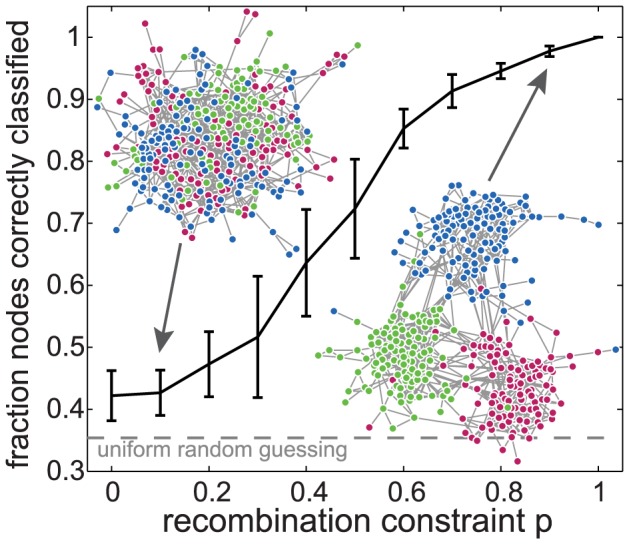
Performance on synthetic data. We validate our method's ability to detect constraints on recombination by testing it on synthetic data with known structure. Sequences were generated at random and divided into three communities, after which 1000 recombination events were simulated, described fully in S2. For each recombination event, the two sequences were forced to be chosen from the same community with probability *p* or were selected uniformly at random with probability (1*-p*). As the probability that recombination is constrained to within-community is varied from no constraint (*p* = 0) to strict constraint (*p* = 1), the ability of our method to correctly classify sequences into one of three communities increases from very poor to perfect. The connected line shows the mean of 25 replicates, with whiskers indicating ± one standard deviation. Two example networks are shown for *p* = 0.1 and *p* = 0.9. The dashed line indicates the accuracy of guessing communities uniformly at random, which is slightly larger than 1/3 as explained in S2. Networks are displayed using a force-directed algorithm that allows a system of repelling point-charges (nodes) and linear springs (links) to relax to a low-energy two dimensional configuration, allowing for visualization of network communities.

In order to confirm that our method is able to correctly recover recombinant communities, we validate it on synthetic data by creating sequences with varying constraints on recombination between predefined groups. We begin by creating amino acid sequences at random from an empirical amino acid frequency distribution and separating them arbitrarily into three groups. Then, we simulate recombination events in which two parent sequences recombine to produce a child sequence, inheriting the group label of one of its parents. We first choose a parent uniformly at random from the population. Then, with probability *p*, the other parent is chosen from the same group and with probability (1*-p*) the other parent is chosen uniformly at random. As *p* is increased from zero to one, the rate of inter-group recombination goes to zero; the communities within the networks produced by applying our sequence analysis method to the synthetically recombined sequences become more well defined, and the method becomes increasingly accurate in correctly classifying nodes. As shown in Figure 3, our method is able to recover recombinant communities perfectly in the presence of strong constraints, and performs only slightly better than random guessing when there are no constraints, as expected. Details of the validation process are found in SI3.

## Results/Discussion

### Discordant patterns of recombination preclude a consensus network

The characteristics and community structures of HVR networks were diverse. Nine HVRs were found in the 307 DBLα sequences [18], using HVR detection parameters of *G = 8* and *H = 6*. These parameters were chosen based on the previously described model for false positive links (SI2), and HVR boundaries were not dramatically affected by small changes to these parameters. The nine HVRs found here corresponded partially to previously identified variable regions over all DBL domains [33]. Since HVRs are by definition highly variable, they consist of mostly gap insertions—this diversity is highlighted by the fact that when gaps were removed after HVRs were identified, sequences shrank by 57% on average. Noise thresholds were computed as shown in Figure 2A such that, in expectation, 10 links (0.02%) or less are false positives, yielding cutoff lengths of 5, 6, or 7 amino acids, varying by HVR. Each HVR showed a wide range of sequence lengths, and HVRs differed widely from each other in median sequence length. HVR lengths, noise cutoffs, and the percentage of links retained for community detection are found in Table 1. The fraction of links above the length cutoffs varied by HVR, shown in Figure 2B and Table 1. For HVRs 2–4, removal of links below the cutoff fragmented the network and in such cases community structure cannot be inferred. Figure 2C illustrates graphically that there did not exist a threshold that both preserved a large connected component and met our requirements for a low false positive rate for HVRs 2–4.

**Table 1:** Summary statistics for DBLα HVRs.

HVR	1	2	3	4	5	6	7	8	9
**Med. length (min-max)**	28 (22–48)	11 (8–17)	6 (4–9)	12 (6–15)	42 (30–57)	29 (25–47)	37 (16–40)	35 (21–49)	39 (8–76)
**Length incl. gaps**	88	27	17	28	106	91	54	68	92
**Noise cutoff**	6	6	5	5	7	6	6	7	6
**% significant links**	6.0	3.1	1.5	2.6	5.8	6.9	24.9	8.4	16.1

**Figure 4:**
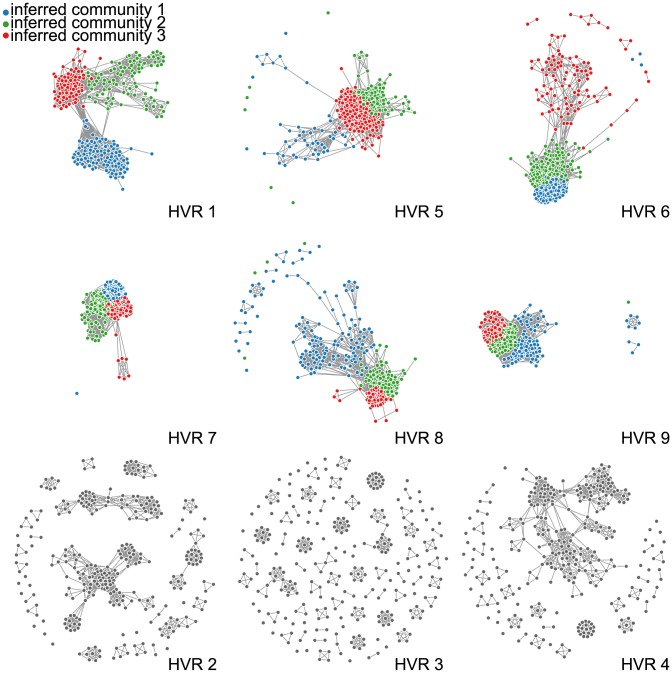
Nine HVR networks colored by inferred communities. DBLα HVR networks show a wide range of characteristics, including community size, number of components, and number of links. Nodes in each HVR are colored according to the best three-community partition identified by the inference algorithm (see text). (Identical networks colored by upstream promotor are found in Figure S2.) HVRs 2–4 are sufficiently fragmented that block model inference cannot be trusted. An interactive version of this figure with varying communities and node labels may be found at http://danlarremore.com/var. Networks are displayed using a force-directed algorithm that allows a system of repelling point-charges (nodes) and linear springs (links) to relax to a low-energy two dimensional configuration, allowing for visualization of network communities.

**Figure 5:**
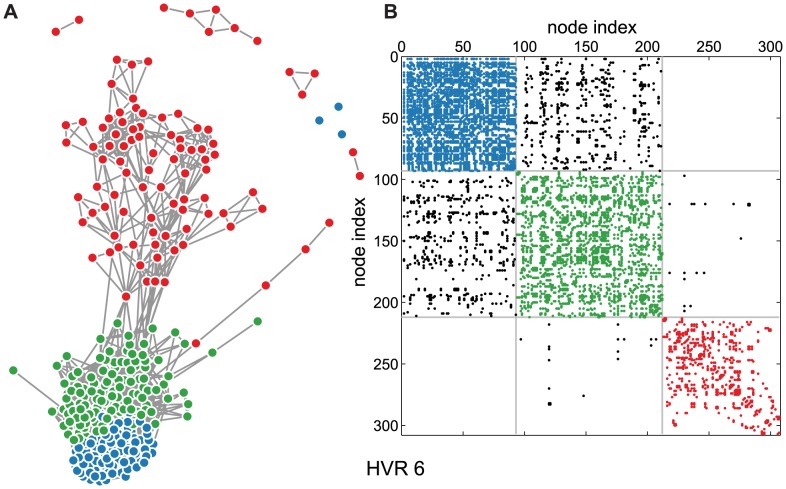
Stochastic block modeling identifies network communities. HVR 6 is shown in two forms, colored according to the best partition into three communities. (A) A force-directed visualization of the network with the identified communities labeled by color. (B) Adjacency matrix in which the ordering of rows and columns has been permuted to match the inferred communities. Diagonal colored blocks are within-community links, and off-diagonal blocks are between-community links. The matrix is shown symmetrically to aid the eye.

Each remaining HVR network had identifiable communities, examples of which are illustrated in Figures 4 and 5. This is consistent with our previous network analysis [13], but provides greater resolution and statistical certainty that these communities represent genuine constraints on recombination. However, the membership lists of communities derived from different HVRs matched each other for only 38% of nodes on average. In addition to having widely varying community structures, HVR networks also differed from each other in component size and number of components illustrated in Figure 4 and tabulated in Table 2. Networks are visually illustrated in Figure 4 and the number and size of components are given in Table 2. Regardless of the number of communities detected, communities corresponded poorly to each other across HVRs.

**Table 2:** Summary statistics for DBLα HVR networks.

HVR Network	1	2	3	4	5	6	7	8	9
**N. components**	1	39	106	51	10	8	2	20	4
**Largest comp. (307)**	307	112	18	183	298	291	306	273	293

**Figure 6:**
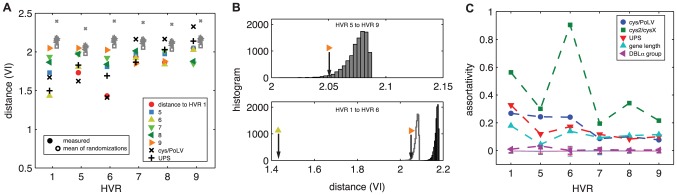
Community structures vary across HVRs. (A) Variation of information (VI) measures the distance between two different partitions on the same set of nodes (two different sets of community assignments). For a given HVR we compute pairwise VI distances to recovered communities of other HVRs (colored symbols), cys/PoLV (+), UPS classifications (x), and to those in a null model (grey open symbols). Uncertainty in VI measurements is discussed in detail in Text S4. (B) HVRs 1 and 6 are close to each other, indicating that their communities strongly match each other. Histograms show the distributions of VI distances for one partition and 10,000 randomizations of the other. Top, the measured distance between HVRs 5 and 9 falls within the distribution of randomizations, indicated by the arrow. Bottom, the measured distance between HVRs 1 and 6 falls well outside the distribution of randomizations, indicated by the left arrow. For contrast, the silhouette of the top histogram is reproduced. (C) Most HVRs show moderate but positive levels of assortativity [45], the tendency for nodes with similar labels or values to be connected. Assortativity varies by HVR and by label (symbols). For all cases except DBLα classification (DBLα0, DBLα1, DBLα2), assortativity was significantly higher than expected by chance. Solid lines with whiskers show mean assortativity ± one standard deviation for 10,000 randomizations of labels. Z-scores may be found in Figure S5A.

HVR networks lack consensus. Traditional phylogenetic approaches often produce a consensus tree that reflects the most likely evolutionary trajectory of a particular gene. However, if patterns of recombination for two HVRs are relatively independent of each other, we expect the communities of one HVR network to match the communities of the other only to the extent they match by chance. In contrast, HVR networks with similar recombinational constraints will have common community structures. In order to quantify the distance between community assignments, we use the variation of information statistic [40], which is a distance metric on partitions. A small value indicates that two partitions are “close” to each other, such that the composition of one is highly correlated with the composition of the other. Figure 6A shows the pairwise distances between the inferred communities for *k = 3* and partitions defined by UPS and cys/PoLV classifications, across HVRs. (Plots for all values of *k* are in Figures S4 and S5B.)

In general, the communities of different HVRs were surprisingly dissimilar, except HVRs 1 and 6, and, to a lesser extent, HVRs 1 and 5. We compared the observed distances to an estimated distribution of pairwise distances for a null model in which we held one partitioning constant and computed distances for 10,000 random permutations of the other, for each pair, shown as grey symbols in Figure 6A and converted to z-scores in Figure S5B. Two examples of the randomized distributions are shown in detail in Figure 6B: the comparison of HVR 5 with HVR 9, and the comparison of HVR 1 with HVR 6. While the distance between HVRs 5 and 9 is smaller than the expected value of a random permutation (top subplot) it is significantly closer to its expected value than HVRs 1 and 6 (bottom subplot). We estimated statistical uncertainty in these measurements and found that in all cases, standard deviations were *O(10^−2^)*, much smaller than the size of colored symbols plotted in Figure 6A. However, we note that this estimate is one of many possible measures of statistical uncertainty for network parameters, each of which is flawed in some way, which we discuss fully in Text S4.

Although no pair of community assignments is farther apart than expected at random, most other community assignments are only very weakly similar to each other. The fact that individual HVRs feature clear community structure implies that there are evolutionary constraints on recombination; yet comparisons of community structure between HVR networks reveal only slightly more similarity than random, suggesting that recombinational constraints at different positions are almost completely independent of each other. Thus, variable selection pressures can be accommodated even within a single DBLα domain (distances are shown as a heatmap for all pair-wise comparisons in supplemental Figure S4). These patterns suggest that mosaic sequences behave as dynamic modules that can be shared among genes relatively intact, with conserved inter-mosaic regions acting as alignment guides in the recombination process. A key finding of this analysis, therefore, is that the relative independence of different HVR networks precludes the use of “consensus” approaches sometimes used to combine trees; HVR networks were sufficiently different that they must be analyzed independently.

### Relationship between HVR communities and previous classification systems

In the absence of tools capable of handling extremely high rates of recombination, *var* genes have been variously classified by their domain structure and gene length, sequence characteristics, upstream promoter regions (UPS), position within the chromosome, and direction of transcription [25], [29]–[32], [40], [41]. However, the correspondence of these groups, which only partially overlap, remains ambiguous. As increasing volumes of *var* gene sequence data are produced from studies in the field, understanding how best to resolve and refine these approaches will be key to interpreting study outcomes. We compared previous classification systems, as well as each parasite genotype, to communities within each HVR network.

Individual parasite *var* repertoires reflect population-level diversity. Examining the sequences of individual parasites, we found that each of the seven parasites' *var* sequences are evenly spread through the clusters of the network, rather than forming genome-specific communities (Figure S1), consistent with previous studies [10]. Regardless of how many communities *k* we choose, each parasite had at least one sequence in each recovered community, showing that a single parasite is not confined to an identifiable genotypic community but instead has samples of all major communities that we identified. This corroborates previous research showing that *var* genotypic diversity within a single parasite is as high as the diversity of the parasite population [20]. This pattern is also consistent with theoretical work has shown that selective pressure on the *var* genes should create parasites with as wide a variety of genotypes as possible for immune evasion, while still preserving enough structure for adhesion and sequestration [42]. Thus, each *P. falciparum* genome contains an antigenic repertoire that is effectively sampled from the diversity of the global pool of *var* genes.

**Figure 7:**
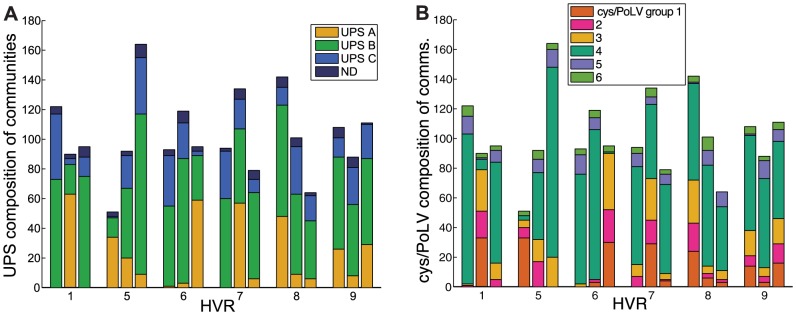
Correspondence of network communities to existing classifications. Bars show the UPS group (A) and cys/PoLV group (B) composition of each of the three recovered communities. HVRs 1 and 6–8 show one community each in which a vast majority of UPS A sequences are found. HVR 6 shows one community in which a vast majority of group 1, 2, and 3 sequences are found, which are all characterized by having only two cysteines in HVRs 5 and 6.

While HVR networks tend to differ from each other, their communities correspond to known upstream promoter sequence (UPS) groupings and *var* gene length. Upstream promoter sequences were previously categorized as UPS A to UPS E or Not Determined (ND) [18]. DBLα with UPS D were not present in the 307 sequences examined. The inferred communities in HVRs 1 and 6–8 place nearly all UPS A sequences together, plotted in Figure 7A. The remaining communities comprise a mix of UPS B, C, and ND sequences. This implies that recombinant mixing of UPS A with B or C is comparatively rare, leading to the hypothesis that three ND sequences could be classified as UPSA: *IT4var24*, *PF07_0048*, and *IT4var51*. Furthermore, we found no evidence for a strong separation of *var* genes into distinct groups for UPS B and C. Although UPS C genes are all found proximate to the centromeres of *P. falciparum* genomes, recombination appears to occur frequently between centromeric and subtelomeric UPS B genes, consistent with studies of chromosomal positioning during ectopic recombination [14], [43], but not yet observed *in vitro* [44].

Using the variation of information distance measure [40], Figure 6A shows that the community structures of HVR 1 and UPS communities are particularly close. This is reinforced by a measurement of assortative mixing by label [45], shown in Figure 6C, a measure of correlation among node labels that takes into account the connections of the network and may be computed without any community assignments. In particular, this reflects the fact that in HVR 1, many UPS A nodes tend to link almost exclusively to other UPS A nodes. Plots of all HVR networks colored by UPS group are found in Figure S2. We also found positive assortative mixing by *var* gene length (including all NTS, DBL, CIDR, and ATS domains) implying that genes of the similar length tend to link to each other, consistent with dynamical models of *var* gene evolution [42] (Figure 6C). Thus, both UPS group and gene length may play roles in constraining recombination, suggesting that future models of evolution and recombination must reproduce these results.

We confirmed and extended the Cys2/Cys4 and PoLV classification schemes which previously identified and analyzed patterns within the DBLα domain [10], [46], [47]. In an alignment-free study of short tagged sequences corresponding to HVRs 5 and 6 from Kilifi, Kenya, it was found that sequences may be classified based on the number of cysteine residues present in HVR 6, and that more severe disease phenotypes were correlated with the presence of only two cysteines [10], [46]. These sequences are referred to as “cys2” sequences, and others, most of which have four cysteines, are referred to as “cysX.” The cys2/cysX classifications can be further classified according to a set of mutually exclusive sequences motifs called positions of limited variability (PoLV), splitting cys2/cysX categories into six cys/PoLV groups [10], [47]. Since the HVR network approach creates links based on shared block structure, it captures cys2/cysX and cys/PoLV categorizations. Unsurprisingly, this is reflected most strongly in HVR6 (Figures 6A and 6C), but is also present in HVR1 whose nodes are strongly assortatively mixed by cys/PoLV group (Figure 6C), corroborating the result that HVRs 1 and 6 are structurally similar. There exists one community in HVRs 1, 6, and 5 that contains most of the cys2 sequences (Figure 7B). The correspondence of network structures with previous phenotype-associated sequences also suggests that the HVR network approach may be extended to map genotypic patterns to clinical phenotypes when applied to an appropriate data set that includes expression data and clinical information.

The DBLα domains we use here were previously analyzed and categorized extensively using tree-based methods, revealing many new subclassifications of the previously identified DBLα0 and DBLα1 [41], [43] as well as a new classification, DBLα2 [18]. We examined HVR networks for evidence of strong associations between network community structure and subclassifications, but found that only DBLα1.3 sequences had a strong tendency to link to other sequences from the same subclassification, particularly in HVRs 7, 8, and 9, where they formed cliques on the periphery of the networks. While the sequence differences of the DBLα1.3 subclass have already been noted [18], we also find that the DBLα0.3 subclass tended to link to each other in HVRs 1 and 8, though not in a clique. No strong correlations were found between network structures and the broader classes of DBLα0, DBLα1, and DBLα2, and networks were not strongly assortative by DBLα class, as shown in Figure 6C. This is perhaps unsurprising, given the different focus of our method, but it highlights the fact that any classification of recombinant genes that is based on a tree-like phylogeny may not be informative about the process of recombination.

### *Var* gene micromodularity facilitates diversity generation without loss of function

**Figure 8:**
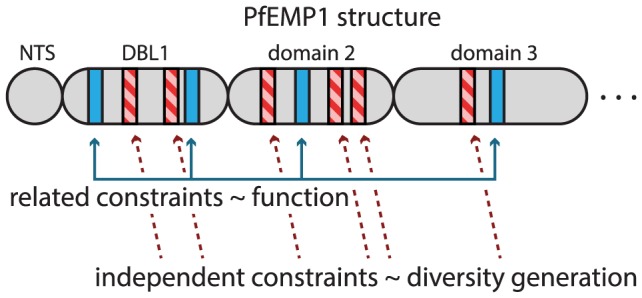
Schematic of HVR hypothesis. Each HVR of DBLα is highly recombinant, yet recombinational constraints, as revealed by network community structure, vary by HVR. We find that some are strongly related (HVRs 1 and 6) while others are independent (HVRs 5 and 9). This suggests that those HVRs that diversify and recombine under constraints that are independent of each other may have the primary purpose of immune evasion by diversity generation (red stripes). Those HVRs whose recombinational constraints are related to each other may recombine only under functional constraints (blue). Independent or related constraints may also be found in other domains throughout the larger PfEMP1 molecule.

Our approach highlights and analyzes the within-domain modularity of DBLα. *Var* genes are by definition modular, with variable numbers and types of DBL and CIDR domains clustered into larger groups that primarily correspond to UPS A and UPS B or C. We have shown that in addition to modular domains that can be shuffled between loci, there are also relatively regularly spaced modular mosaics within the DBLα domain that are shuffled, under constraints, between *var* genes. Previous studies have suggested that the conserved blocks in DBL domains may provide structural support for the protein while the variable regions in between are loops in the protein designed specifically for antigenic variation under diversifying selection [17], [33]. Here we offer a more nuanced hypothesis, shown schematically in Figure 8: HVRs under related recombinational constraints may have important functional roles in the PfEMP1 molecule, while other HVRs may exist primarily for purposes of antigenic variation.

We find that HVRs 1, 6, and to a lesser extent 5, have similar non-random network community structure that corresponds strongly with structural amino acid residues and classification systems with known associations with severe disease [47]. These regions of the domain may therefore be functionally constrained and play a specific role in binding. Interestingly, HVRs 5 and 6 correspond to the short tag sequences that have previously been amplified from field isolates. If our hypothesis is correct, it would explain why the clustering of these sequence tags exhibit meaningful associations with disease outcome [46], [48]. The remaining HVRs 7–9, have highly heterogeneous community structures that bear almost no relation to each other, suggesting that their primary role is in the generation of diversity for the purpose of immune evasion.

Having both correlated and uncorrelated recombinational constraints across multiple HVRs thus provides at least two important evolutionary benefits to the parasite: i) individual mosaics that are functionally important can retain their function without compromising the generation of diversity across the rest of the domain, and ii) recombination could produce variants through different combinations of modules more rapidly than mutation or random recombination, and without risking the recombinational equivalent of error catastrophe that occurs in systems with very high mutation rates [49]. In other words, the parasite population may be rapidly shuffling ancient sequence mosaics into new combinations across some HVRs, while also preventing the degeneration of structurally important regions of the protein that are involved in binding. The population of genes may thereby balance a need for new diversity with functional requirements.

The varying correspondence of HVR communities with previously defined *var* gene groupings implies that the different classification schemes complement each other, providing insights into different aspects of *var* gene evolution, likely representing nested or hierarchical recombinant clusters. We measure the extent to which previously defined groupings are reflected in the links of our networks using assortativity, shown in Figure 6C. Importantly, the DBLα group assortativity is much lower than all the others, demonstrating further that while there are clear structures in HVR networks, they are not the same as the classifications based on trees. Such tree-based classifications explain the development of large-scale structure over the relatively longer timescales of mutation, whereas communities detected within recombination networks here shed light on functional or even mechanistic constraints on recombination occurring more recently.

### Conclusions

The method presented here can accurately extract block-sharing networks from the most highly recombinant regions of protein sequences. While a multiple-alignment is used to identify HVRs (Figure 1A), we find that HVR boundaries are robust to changes in alignment parameters, and the remaining steps of our network extraction process (Figures 1B–D) are entirely alignment-free. Since the structure of such networks reveals patterns of recombination, strong communities within networks are indicative of functional or evolutionary constraints on recombination within the underlying population. By applying this technique to the DBLα domain of *var* genes we find that different locations of the domain produce different communities. Were the constraints identical in each domain, we would expect network communities to be similar to each other and exhibit small variation of information distance. The fact that network communities differ therefore indicates that while constraints exist at each location in the domain, they also vary by location. We suggest that this lack of correlation between constraints allows the DBLα domain to possess exponentially more complexity while simultaneously remaining functional, avoiding recombinant error catastrophe despite extremely high rates of recombination.

The combination of principled and alignment-free network construction methods with state-of-the-art generative models for community detection may open the door to new research areas, linking evolution, function, and clinical phenotypes in a range of genetically diverse pathogens. While we demonstrate our method using DBLα, it could also be applied to other highly recombinant *var* domains, other *P. falciparum* genes such as the *rif* and *cirs* families, or other pathogens, such as HIV, the pneumococcus, or trypanosomes. Methodologically, extensions include the development and application of stochastic block model community detection in weighted networks, treating the separate HVR networks as a single multiplex network [50], and generalizing the current process to inexact sequence matches.

## Supporting Information

Figure S1 **Diversity of individual parasites.** Sequences from a single parasite (large nodes) are distributed evenly throughout the recovered groups. Thus, the diversity within a single parasite is comparable to the diversity of the entire network. (A) HVR 1 with four inferred communities, highlighting the DD2 parasite, and (B) HVR 6 with three inferred communities, highlighting the RAJ116 parasite provide two representative examples. Figures depict differing numbers of communities to show that single parasite genomes are distributed throughout network communities, even as the number of communities increases. An interactive version of this figure may be found at http://danlarremore.com/var. Networks are displayed using a force-directed algorithm that allows a system of repelling point-charges (nodes) and linear springs (links) to relax to a low-energy two dimensional configuration, allowing for visualization of network communities.
(EPS)

Figure S2 **Nine HVR networks colored by UPS grouping.** DBLα HVR networks are colored by Upstream Promoter Region (UPS) with categories of A,B,C, or Not Determined. An interactive version of this figure with varying communities and node labels may be found at http://danlarremore.com/var. Networks are displayed using a force-directed algorithm that allows a system of repelling point-charges (nodes) and linear springs (links) to relax to a low-energy two dimensional configuration, allowing for visualization of network communities.
(EPS)

Figure S3 **Phylogenetic trees of DBLα sequences.** (A) A SplitsTree diagram displays a phylogenetic tree in which ambiguous or recombinant sequences are linked with a cross-link, as described in S3. The presence of many cross links and very little reliable tree structure demonstrates the need for tools beyond traditional phylogenetic trees and networks. (B) A phylogenetic tree was constructed using RaxML 7.0 as described in S3, and color-coded by UPS group. While the tree classifies nearly all UPSA sequences together, poor bootstrap values indicate that this phylogeny has low statistical reliability.
(EPS)

Figure S4 **Pairwise distances between HVR communities.** Similarities in community structures are measured here by the variation of information (VI) metric [40], pairwise for each HVR and for *k* = 3, 4, 5, and 6 communities, as well as cys/PoLV and UPS classifications. Small distances on the ±6-, ±12-, and ±18-diagonal stripes indicate that each HVR is more similar to itself (regardless of the number of communities inferred) than it is to any other HVR. This indicates that HVR networks do possess distinct community structures, but that such structures vary widely by HVR. A similar figure showing z-scores of VI from randomization of labels is included in Figure S5B. VI values from the regions in the black boxes are displayed graphically in Figure 6A. Accordingly, HVRs 1 and 6 are much closer than any other pair of partitions of two different HVRs for a variety of *k* values.
(EPS)

Figure S5 **Node label randomization tests.** Results from randomization trials show that the assortativities shown in Figure 6C and some of the distances shown in Figure S4 are statistically significant, because the probability that the null model would yield as small or smaller distances is minute. (A) Each point is an assortativity z-score calculated from 10,000 randomizations of a network's node labels, showing that network assortativities by label and gene length reflect a preference of sequences to recombine with other similar sequences. (B) Each point is a VI z-score from 10,000 randomizations of node labels. As in Figure S4, HVRs are more similar to themselves (with any number of communities) than they are to each other. HVRs 1 and 6 are again shown as more similar to each other than other pairs, followed closely by HVR1 and UPS, and HVR6 and cys/PoLV communities. It is important to note, however, that randomized variation of information distances are not normally distributed, and so these z-scores should not be interpreted as such.
(EPS)

Figure S6 **Choice of link noise threshold (** **Figure 2** **with all HVRs shown).** While Figures 2A and 2C illustrate the method by which link noise threshold is selected, we excluded other HVRs to provide a more legible and understandable figure. Here we show all nine HVRs. (A) The probability of two sequences sharing a block while not actually being related decreases rapidly for all HVRs. We exclude all block lengths falling above a noise threshold (grey region) and choose the next largest block length as the appropriate minimum block length (squares). (B) HVR networks fragment as block length threshold is increased and more links are discarded. Grey points indicate cases that are prohibited due to the cutoff shown in Figure S6A, while colored points are permissible. However, points in the grey shaded region are prohibited due to fragmentation of the network. This precludes analysis of HVRs 2–4, for which no suitable threshold exists that is both low-noise and unfragmented, indicated by red circles.
(EPS)

Text S1 **Null model for sharing of protein sequence substrings.** A detailed description of the approximation of the rates at which sequences share substrings of varying lengths purely by chance.
(PDF)

Text S2 **Validation on synthetic data.** A detailed description of the procedures used to generate and analyze synthetic data for the purposes of validation.
(PDF)

Text S3 **Detailed description of phylogenetic analyses and software.**
(PDF)

Text S4 **Uncertainty in measurements of variation of information from inferred partitions.** A detailed discussion of the current state of the literature in assessing the uncertainty in community detection methods, variation of information, and other network statistics.
(DOCX)
