# Supplementary material for: A Network Approach to Analyzing Highly Recombinant Malaria Parasite Genes
Source: PLoS Comput Biol. 2013 Oct 10;9(10):e1003268. doi: 10.1371/journal.pcbi.1003268 (PMC3794903; doi:10.1371/journal.pcbi.1003268)
Supplement: Text S1 — Null model for sharing of protein sequence substrings. A detailed description of the approximation of the rates at which sequences share substrings of varying lengths purely by chance. (PDF) [file pcbi.1003268.s007.pdf]

Text S1: Null model for sharing of protein sequence substrings

Creating networks requires comparing many pairs of amino acid sequences. For each pair, we seek the length of the longest substring shared by both sequences. Some sequence pairs may have zero amino acids in common, in which case the length of the longest shared substring will be zero. However, most pairs of sequences will share a common substring that is at least one amino acid in length. How long must a shared substring be in order to be retained as a meaningful link in the network? Here, we compute the probability that a shared substring of a particular length arises simply by chance in order to allow a principled method by which to choose a threshold for link retention.

Suppose that we construct two sequences called n and m, of length $|n| = N$ and $|m| = M$, respectively, by drawing amino acids one-by-one from a replenishing urn from which each acid i will be drawn with probability p_i. What is the probability that they share one or more substrings of length L?

For convenience, we define $\mathcal{S}^L \equiv$ the set of all possible amino acid sequences of length L. Since there are 20 amino acids derived from nucleotide codons, $|\mathcal{S}^L| = 20^L$. We enumerate these by i, so that $\mathcal{S}^L = \{s_i\}_{i=1}^{20^L}$

$$\begin{aligned}
\text{Pr(at least one shared string of length } L) &= 1 - \text{Pr(no shared strings of length } L)\\
&= 1 - \prod_{s_i \in \mathcal{S}^L} \text{Pr(string } s_i \text{ is not shared)}\\
&= 1 - \prod_{s_i \in \mathcal{S}^L} 1 - \text{Pr(string } s_i \text{ is shared at least once)}\\
&= 1 - \prod_{s_i \in \mathcal{S}^L} 1 - \text{Pr(string } s_i \text{ is in sequence } n \text{ AND in sequence } m)\\
&= 1 - \prod_{s_i \in \mathcal{S}^L} 1 - \text{Pr(string } s_i \text{ is in sequence } n) \times \text{Pr(string } s_i \text{ is in sequence } m) \quad (1)
\end{aligned}$$

We pause here for a side calculation, noting that there are $N - L + 1$ different ways to position a string of length L in a sequence of length N:

$$\begin{aligned}
\text{Pr(string } s_i \text{ is in sequence } n) &= \text{1- Pr(string } s_i \text{ is not in sequence } n \text{ anywhere)}\\
&= 1 - \prod_{j=1}^{N-L+1} [1 - \text{Pr(string } s_i \text{is found in position } j)]\\
&= 1 - [1 - \text{Pr(string } s_i \text{is drawn from urn)}]^{N-L+1} \quad (2)
\end{aligned}$$

with the last step coming from the assumption of homogeneity and independence of the probabilities at different positions of the sequence, and therefore all $N - L + 1$ positions may be treated equally as being drawn from an urn IID. This assumption of independence means that we will overestimate the probability of sequence sharing. Therefore, after this point, the calculation yields an upper bound for probability. Substituting Eq. (2) into Eq. (1) yields

$$\text{Pr(at least one shared string of length } L) \leq 1 - \prod_{s_i \in \mathcal{S}^L} \left\{ 1 - \left[1 - (1 - \text{Pr}(s_i))^{N-L+1} \right] \times \left[1 - (1 - \text{Pr}(s_i))^{M-L+1} \right] \right\}. \quad (3)$$

When calculating $\text{Pr}(s_i)$ in practice we model strings as being drawn $i.i.d.$ from a multinomial distribution in which each of the amino acids i has probability p_i, which we set to the maximum likelihood value f_i/T, where f_i is the empirical frequency of acid i and T is the total number of acids observed. Since amino acid composition varies with HVR, this approach takes into account the composition of each HVR separately. Similarly, the lengths of sequences in each HVR are different as shown in Table 1. To simplify computation, we let $N = M$ in Eq. (3), yielding

$$\text{Pr(at least one shared string of length L)} \leq 1 - \prod_{s_i \in \mathcal{S}^L} \left\{ 1 - \left[1 - (1 - \text{Pr}(s_i))^{N-L+1} \right]^2 \right\}. \quad (4)$$

We use Eq. (4) to compute each curve in Figure 2A, setting N to the median length of each HVR (Table 1), allowing a translation between an expected number of false positives and a length threshold below which links should be discarded. Since it is an upper bound, strictly adhering to the threshold that it prescribes will result in fewer false positive links, but may also result in fewer true positive links.
