# Supplementary material for: A Network Approach to Analyzing Highly Recombinant Malaria Parasite Genes
Source: PLoS Comput Biol. 2013 Oct 10;9(10):e1003268. doi: 10.1371/journal.pcbi.1003268 (PMC3794903; doi:10.1371/journal.pcbi.1003268)
Supplement: Text S2 — Validation on synthetic data. A detailed description of the procedures used to generate and analyze synthetic data for the purposes of validation. (PDF) [file pcbi.1003268.s008.pdf]

Text S2: Validation on synthetic data

In order to demonstrate that the community structures found in real networks may correspond to real constraints on recombination, we create synthetic amino acid sequences and allow them to recombine under various levels of a recombination constraint and measure the accuracy of our method. The specific procedures followed were: (i) Create sequences: choose an empirically measured distribution of amino acid frequencies from an existing HVR. Using the empirical distribution as the parameters of a multinomial distribution, draw amino acids IID in order to generate 60 sequences, each of length 30. Arbitrarily separate the sequences into three groups of 20 sequences each, labeled A, B, and C. (ii) Simulate recombination: uniformly at random, choose an existing sequence as mother, noting her group. With probability p, choose a father uniformly at random from the same group as the mother; with probability $(1-p)$ choose the father uniformly at random from all sequences. Mimic a gene conversion by choosing uniformly at random a block of length a from the mother and length b from the father, creating a child that is a copy of the mother, but with the father's block replacing the mother's. Here we choose a, b ~Unif$[8, 12]$. Place the child into the same group as the mother. Repeat this step 1000 times, each time placing the child into the pool of sequences from which mother and father may be selected, resulting in a total of 1060 sequences. Discard the original 60 progenitor sequences. (iii) Create networks. Forgo the step of identifying HVRs—we assume that our synthetic data comes from a single HVR. (iv) Find communities using the degree-corrected stochastic blockmodel with $k = 3$ communities, classifying nodes as community X, Y, or Z. (v) Measure accuracy as the fraction of nodes that are matched between X, Y, Z, and A, B, C. There are six possible pairing sets of the original communities and the detected communities, so we measure accuracy as the pairing set that results in the maximal value. This means that simply guessing communities uniformly at random would result in accuracies slightly higher than $1/3$ because the accuracy-maximizing pairing set is bounded from below by $1/3$ and is often larger, stochastically. (vi) Measure accuracy for 25 replicates at various values of p. We note that when $p = 0$ accuracy is still slightly better than random guessing due to weak network structures induced by heredity patterns.
