# Supplementary material for: A Network Approach to Analyzing Highly Recombinant Malaria Parasite Genes
Source: PLoS Comput Biol. 2013 Oct 10;9(10):e1003268. doi: 10.1371/journal.pcbi.1003268 (PMC3794903; doi:10.1371/journal.pcbi.1003268)
Supplement: Text S3 — Detailed description of phylogenetic analyses and software. (PDF) [file pcbi.1003268.s009.pdf]

Text S3: Phylogenetic analyses

Protein sequences were aligned using MUSCLE v3.8 (PMID: 15034147 [1]). Different models of sequence evolution were then evaluated using ProtTest v3.2 (PMID: 21335321 [2]); based on the Bayesian information criterion, this found the WAG matrix combined with a gamma rate distribution and a calculation for the proportion of invariant sites to be the most appropriate evolutionary model. These settings were therefore used to generate a maximum likelihood distance matrix and phylogeny using RAxML v7.0 (PMID: 16928733 [3]), with 100 bootstrap replicates produced to measure support for internal branches. The distance matrix was analysed using SplitsTree v4 (PMID: 9520503 [4]) as a further measure of uncertainty in the phylogeny.

References

[1] Edgar RC (2004) MUSCLE: multiple sequence alignment with high accuracy and high throughput. Nucleic Acids Research 32: 17921797. doi:10.1093/nar/gkh340.

[2] Darriba D, Taboada GL, Doallo R, Posada D (2011) ProtTest 3: fast selection of best-fit models of protein evolution. Bioinformatics 27: 11641165. doi:10.1093/bioinformatics/btr088.

[3] Stamatakis A (2006) RAxML-VI-HPC: maximum likelihood-based phylogenetic analyses with thousands of taxa and mixed models. Bioinformatics 22: 26882690. doi:10.1093/ bioinformatics/btl446.

[4] Huson DH (1998) SplitsTree: analyzing and visualizing evolutionary data. Bioinformatics 14: 6873.
