# Supplementary material for: A Network Approach to Analyzing Highly Recombinant Malaria Parasite Genes
Source: PLoS Comput Biol. 2013 Oct 10;9(10):e1003268. doi: 10.1371/journal.pcbi.1003268 (PMC3794903; doi:10.1371/journal.pcbi.1003268)
Supplement: Text S4 — Uncertainty in measurements of variation of information from inferred partitions. A detailed discussion of the current state of the literature in assessing the uncertainty in community detection methods, variation of information, and other network statistics. (DOCX) [file pcbi.1003268.s010.docx]

**Text S4: Uncertainty in measurements of VI from inferred partitions**

In the main text, a stochastic block model is fit to network data, generating a partition of the network nodes into communities, for each HVR. The distance between partitions, as measured by variation of information (VI), provides an idea of how similar or different community structures are between HVRs. The question of whether or not the difference between certain VI distances is large or small is an important one. If the differences are large, then we can say the pair of partitions really are different from each other, while if the difference is small, we conclude the opposite. However, inference of the parameters of the stochastic block model raises two questions about uncertainty:

1. What is the uncertainty in VI due to uncertainty in the likelihood maximization algorithm?
2. What is the uncertainty in VI due to uncertainty inherent to the likelihood function itself?

The first question is straightforward to address, yet the second is less so; uncertainty of likelihood functions of stochastic block models is the subject of ongoing theoretical research. This supplement provides a more thorough and lengthy discussion of the technicalities involved in answering both.

1. *What is the uncertainty in VI due to uncertainty in the likelihood maximization algorithm?*

The algorithm used to find the maximum likelihood partition is described fully by Karrer and Newman [1] and is similar to the Kernighan-Lin partitioning algorithm:

“Briefly, in this algorithm we divide the network into some initial set of K communities at random. Then we repeatedly move a vertex from one group to another, selecting at each step the move that will most increase the objective function— or least decrease it if no increase is possible—subject to the restriction that each vertex may be moved only once. When all vertices have been moved, we inspect the states through which the system passed from start to end of the procedure, select the one with the highest objective score, and use this state as the starting point for a new iteration of the same procedure. When a complete such iteration passes without any increase in the objective function, the algorithm ends. As with many deterministic algorithms, we have found it helpful to run the calculation with several different random initial conditions and take the best result over all runs.”

Since we initialize this algorithm with a random initial condition, due to roughness of the optimization landscape, a different local maximum may be found each time the algorithm is run. We can therefore calculate uncertainty in VI by the following procedure.

1. Rerun the algorithm *M* times, computing an ensemble of *M* optimal partitions and their likelihoods.
2. Compute VI pairwise between all pairs of optimal partitions, yielding *M^2^* estimates.
3. Compute a weighted average and weighted standard deviation of the ensemble where the weight of each datum is the sum of the two partitions’ log likelihoods.

This procedure is similar to a Bayesian model averaging approach, and allows for a statistically rigorous estimate of the underlying variance of the point estimates of the VI between each pair of HVRs. When we performed this analysis on our data, we found that standard deviations of VI distances were all *O(10^-2^)*, suggesting that the roughness of the optimization landscape has little effect on the VI distances shown in Figure 6.

1. *What is the uncertainty in VI due to uncertainty inherent to the likelihood function itself?*

When data are scalar- or vector-valued this question can be answered in several ways that will provide reliable and statistically principled answers. One is to perform a bootstrap of the original data in order to estimate the bootstrap distribution of the estimated parameter. Another is to examine the local curvature of the likelihood function around the maximum likelihood estimate (MLE). Asymptotically, these approaches converge on the same uncertainty estimates.

However, the data here are not scalar- or vector-valued, but are instead relational, i.e., a network, and their interdependence violates the underlying assumptions of the bootstrap that would otherwise allow it to produce statistically rigorous uncertainty estimates. As a result, a bootstrap on network nodes does not produce an answer to question 2 because bootstrap networks are statistically dissimilar to the empirical data in crucial ways.

For example, a bootstrapped network, whose nodes are resampled from original data with replacement, will have sampled some nodes multiple times and other nodes zero times. Those nodes that are sampled multiple times will form identical cliques with their clones, while nodes that have been sampled zero times may fragment the network into more components than in the original network. Resampled networks tend to have higher clustering coefficients (density of triangles) and occasionally more components as a result. Notably, the goal of this step of uncertainty analysis is to generate a distribution of plausible replicates of the test statistic, so if the resampled data sets are structurally very different from the empirical one, then the estimated distribution is not informative of the uncertainty in the statistic calculated from the original data set.

This may be demonstrated in the case of VI by creating two realizations of the generative stochastic block model, producing two networks with known community structure. Inference of communities using the stochastic block model will produce extremely similar (though not identical, due to noise) partitions, and thus VI will be very small. However, bootstrapping VI in this case will result in a distribution that is comparatively very wide and is also not centered at the empirical value.

Alternatively, one could conceive of bootstrapping VI by resampling edges instead of nodes, yet this, too, does not solve the problem. Edges in recombination networks represent characteristics of the vertices and thus are not independent of each other. Furthermore, the SBM used here is not defined for multi-graphs, which resampling the edges would produce.

Both of these issues point to a fundamental issue with bootstrapping network statistics: individual vertices are not IID draws of some underlying distribution, as in the case for most scalar- and vector-valued data, that can be considered independently of each other—a requirement for the bootstrap to produce consistent estimates. Instead, our networks are highly structured sets of interrelationships.

One may also consider a *leave-k-out* technique (e.g., a statistical jackknife) in which, rather than resampling with replacement as in bootstrapping, *k* sequences are left out. This process has the major advantage of avoiding some of the non-random structural perturbations discussed above. Unfortunately, it has two large disadvantages that make it a non-viable alternative for examining the VI statistic. First, reducing the number of sequences used to construct the network changes the scale on which the VI is calculated—VI is bounded between 0 and roughly the log of the number of vertices—which makes these VIs non-comparable to the empirical value. Second, subsampling presents similar violations of independence assumptions as in the bootstrap, and as a result the nice statistical properties (under IID conditions) of subsampling are not guaranteed.

Rather than a resampling approach, one might attempt to directly measure the local curvature of the likelihood function by Taylor expanding about the MLE. However, this requires a nice analytic form that may be expanded, as well as a likelihood function that is convex, i.e. has a single global maximum. The stochastic block model meets neither of these criteria. A more principled solution may be to sample the local optima of the likelihood function and estimate the variance in the test statistic using this ensemble (as described in the answer to question 1 above), yet this technique may confound likelihood function uncertainty with the uncertainty of the search algorithm.

The conclusion is that uncertainty, as well as model selection, for stochastic block models (and principled inference techniques for networks in general) are not yet well studied enough to provide a definite answer to question 2. The answer to question 1 is optimistic, but we have included the preceding discussion so that readers interested in applying the techniques described here may avoid statistical pitfalls in their work.
